# Exploring Clustered Regularly Interspaced Short Palindromic Repeats-CRISPR-Associated Protein 9 (CRISPR-Cas9) as a Therapeutic Modality for Cancer: A Scoping Review

**DOI:** 10.7759/cureus.64324

**Published:** 2024-07-11

**Authors:** Abirami Balasubramanian, Keerthana Veluswami, Sudipta Rao, Shailesh Aggarwal, Sweatha Mani

**Affiliations:** 1 Internal Medicine, Stanley Medical College, Chennai, IND; 2 Internal Medicine, Jagadguru Sri Shivarathreeshwara (JSS) Medical College, Mysore, IND; 3 Internal Medicine, K.A.P. Viswanatham Government Medical College, Tiruchirappalli , IND

**Keywords:** direct base editing, advanced therapy, cancer immunotherapy, genome editing, oncology, cancer, crispr-cas, crispr cas9

## Abstract

The global burden of cancer and the limitations of conventional therapies highlight the potential of clustered regularly interspaced short palindromic repeats-CRISPR-associated protein 9 (CRISPR-Cas9) in reshaping cancer treatment paradigms. In this review, we have investigated the mechanism of CRISPR, an adaptive immune system in bacteria that enables highly precise gene editing at the molecular level. This versatile tool demonstrates its efficacy in human cancer therapy through gene knockout, metabolic disruption, base editing, screening, and immunotherapy enhancement without affecting normal bodily domains. Despite its superiority over other nucleases like zinc-finger nucleases and transcription activator-like effector nucleases, hurdles such as off-target effects, inefficient delivery of the system to target cells, the emergence of escapers, and the ethical debate surrounding genome editing are discussed. In this article, we have reviewed the promising approaches of CRISPR-Cas9 in cancer treatment while exploring the underlying mechanism, advantages, and associated challenges.

## Introduction and background

Cancer stands as a significant global health challenge, with nearly two million annual diagnoses, making it the second leading cause of death in the United States [1]. The National Cancer Institute has set a goal to achieve a minimum 50% reduction in the overall cancer death rate by the year 2047 while simultaneously enhancing cancer care for all individuals [1]. Cancer is inherently a genetic disease, arising from the gradual accumulation of genetic alterations that disrupt growth signals within the body [2]. Consequently, the management of cancer is said to be a highly complex process [3]. The conventional treatments of chemotherapy and radiation therapy, either alone or in combination, have shown improved survival rates [4], but their effectiveness is tempered by detrimental side effects such as chemotoxicity, nausea, vomiting, diarrhea, oral mucositis, drug resistance, and so on, which can significantly impact daily life [4-6]. Another widely acknowledged modality is surgery [7]. However, it is an invasive procedure and may not be universally accessible, particularly to lower-income households with an increased risk of postoperative complications [8] and recurrence [9]. These limitations impose a significant mental toll on individuals, giving rise to psychosocial concerns [5]. With the surging rates of cancer incidence and associated mortality, there has been a pressing need for a broader range of treatment modalities that offer enhanced prognostic outcomes.

Advancements in science and research have paved the way for groundbreaking modalities that were once considered implausible, helping in the discovery of gene-specific treatments, namely immunotherapy and gene therapy. Immunotherapy harnesses the body’s immune system to target and combat cancer cells in the form of cancer vaccines, checkpoint blockade therapy, and adoptive cell transfer [10]. On the other hand, gene therapy involves the introduction or modification of genetic material to treat or prevent diseases, holding immense promise for personalized and precise cancer treatments [11]. These cutting-edge techniques signify a paradigm shift in the approach to cancer care, offering a highly targeted approach with improved outcomes. However, it is crucial to acknowledge that the satisfactory outcomes were only limited to a subset of cancer types [12]. Moreover, initial attempts at gene therapy raised concerns about insertional mutations [13]. Traditionally, gene delivery to the target tissues relied on viral vectors. Challenges included handling viral vectors due to the risk of vector-related toxicity, high cost, and time-consuming labor-intensive work to reduce this toxicity [14] and adaptive immunity of the body, reducing the clinical response to the viral vectors and ultimately the efficacy of viral vectors [15].

**Figure 1:**
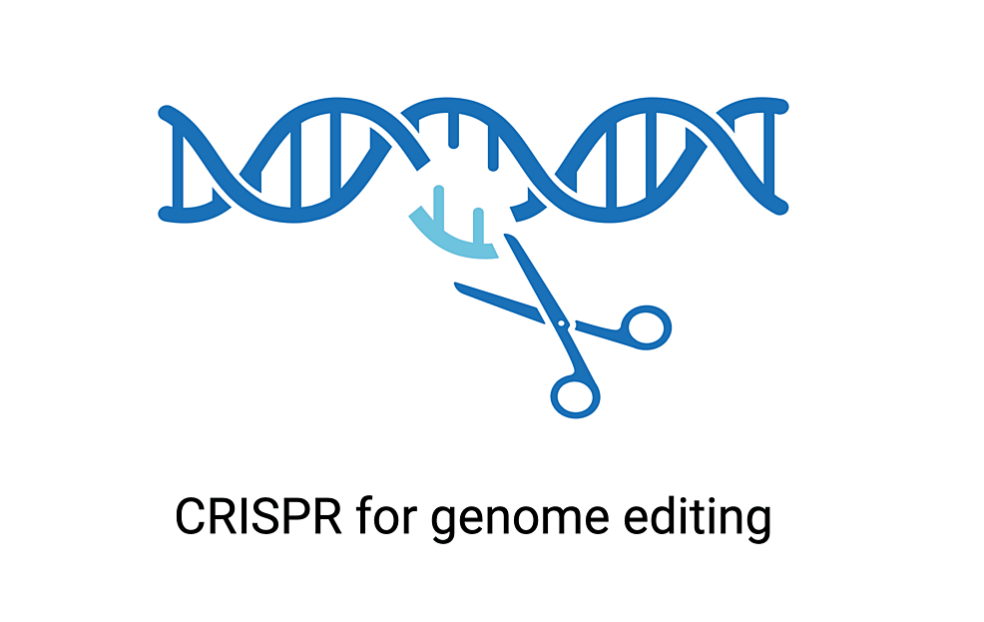
CRISPR for genome editing CRISPR, clustered regularly interspaced short palindromic repeats Created with BioRender.com; Image credit: Abirami Balasubramanian

Overcoming the limitations of gene therapy and allowing a significant shift from merely introducing genes to the art of genome editing instead was the invention of a gene editing tool, namely clustered regularly interspaced short palindromic repeats (CRISPR). As mentioned by Khan et al., CRISPR is a novel family of repetitive DNA sequences that belong to prokaryote genomes that enables precisely editing, adding, or deleting specific DNA sequences with unparalleled accuracy and carries the potential to cure cancer (Figure 1) [16]. Once considered ambitious speculation, genetic manipulation by CRISPR has emerged as a revolutionary approach to fighting diseases, particularly cancer. This paper aims to explore the role of CRISPR as a groundbreaking tool at the forefront of cancer research and its potential to reshape the way we treat cancer.

## Review

Database search strategy

To prepare this review, searches were conducted through PubMed, Google Scholar, and journals indexed in the PubMed database. Keywords used to find pertinent articles included CRISPR, Cas9, genome editing, and cancer. The search strategy involved the use of Medical Subject Headings (MeSH) and the application of Boolean operators (AND, OR) to refine and narrow down the search. An emphasis was placed on recent publications to ensure the review is up to date. The inclusion criteria focused on topic-specific narrative reviews and systematic reviews. The search results were initially screened by title, abstract, and keywords, then the full text was reviewed to extract relevant information from each publication.

Mechanism of CRISPR-CRISPR-associated protein 9 (Cas9)

CRISPR represents a unique natural mechanism embedded within the genome of prokaryotes, both in Gram-positive and Gram-negative bacteria, as well as Archaea [16,17].

These short palindromic repeats play a pivotal component of adaptive immunity that protects prokaryotes from attack by viral DNA, bacteriophages, and plasmids. Adaptive immunity, akin to vaccination in humans, refers to the immunity fostered by the organism after exposure to an antigen [17,18]. This similar mechanism happens in bacteria as well, with the help of unique sequences located in between the palindromic repeats called “spacers.” These spacers, identified through sequence analysis, represent foreign DNA originating from mobile genetic elements such as bacteriophages, transposons, or plasmids that have previously infected the bacteria [18]. This insight further contributed to formulating the theory that such incorporation serves as a crucial component of bacteria’s defense mechanism, aiding in the recognition of foreign elements [18].

Upon attack by a virus, the bacteria respond by incorporating a fragment of the viral DNA into the CRISPR locus, a process that gives rise to CRISPR arrays [18]. This integration serves as a means through which the bacteria retains a memory of the viral infection, similar to memory T cells within the human body [17]. The outcome is an amalgamation of regularly interspaced palindromic repeats derived from bacterial DNA and variable sequences of viral DNA, forming spacers sandwiched in between [17,18].

**Figure 2:**
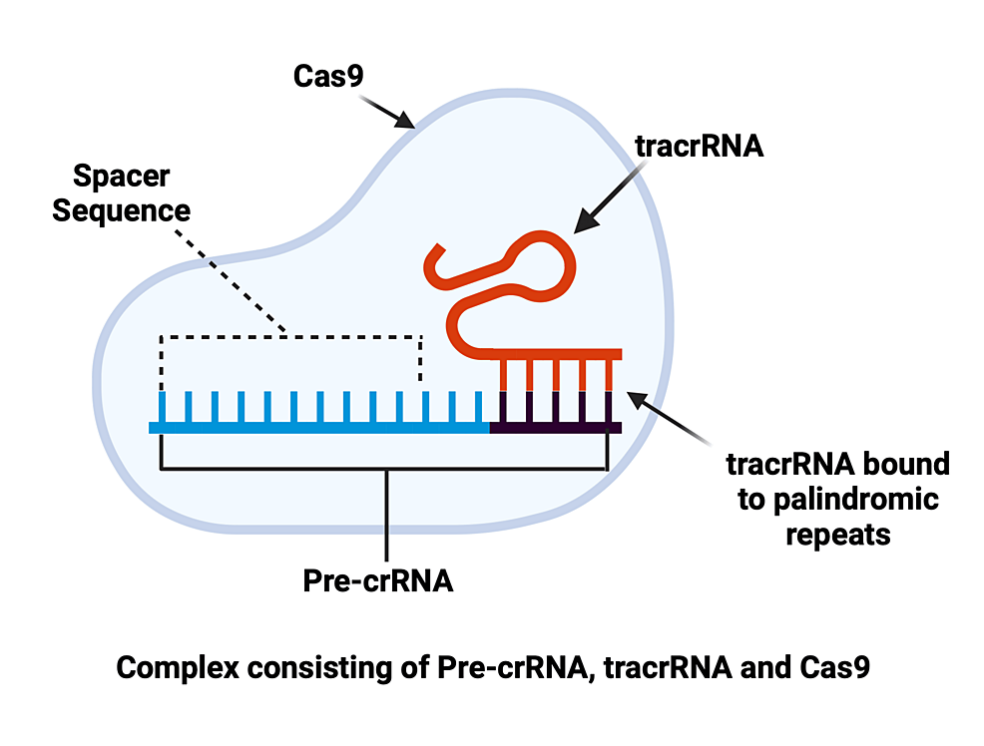
Effector complex comprising pre-crRNA, tracrRNA, and Cas9 nuclease Cas9, CRISPR-associated protein 9 Created with BioRender.com; Image credit: Abirami Balasubramanian

This CRISPR array undergoes transcription, producing CRISPR RNA in the form of pre-crRNA. This process involves Cas9 proteins, which function as molecular scissors that precisely cleave DNA at specific nucleotide linkages [19]. Notably, this Cas9 nuclease has been extensively observed in *Streptococcus pyogenes*, one of the most experimented-on organisms for CRISPR. During this transcription, molecules of tracrRNA, complementary to the palindromic repeats, anneal to them and provide a platform for Cas9 nuclease to bind the target DNA [20]. This now completes a complex comprising pre-crRNA, wherein a palindromic repeat is annealed to tracrRNA, along with the Cas9 protein (Figure 2) [19,20].

Another pivotal enzyme, ribonuclease 3, or RNAse 3, cleaves the strand in between these complexes, generating individual complexes, namely effector complexes [21]. Now these effector complexes play a crucial role in defending against viruses whose genomes produce the crRNA. Upon encountering a section of viral DNA complementary to the sequence of crRNA and recognizing a short, unique sequence in the viral genome known as the protospacer adjacent motif (PAM), the effector complex snips both strands of the DNA, a few bases upstream of PAM, with coordination of the nuclease protein [21]. This neutralizes the virus, rendering it incapable of producing an intact genome for transcription and preventing further virus particle generation. Consequently, infection by that particular virus becomes impossible [19,21].

**Figure 3:**
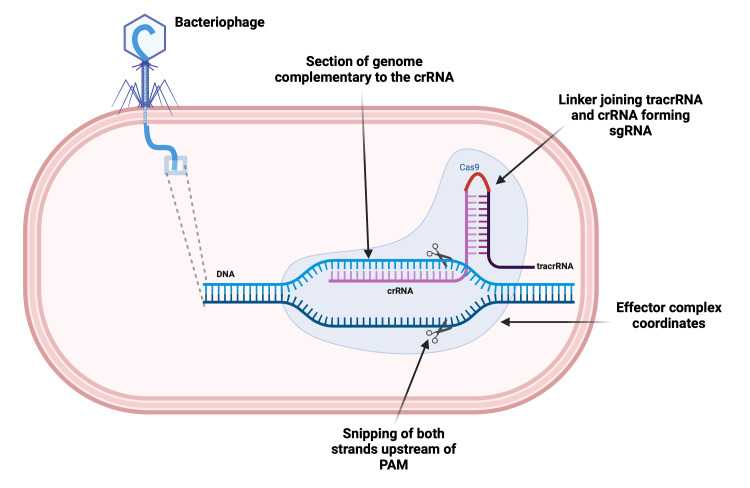
sgRNA with Cas9 forming the effector complex, which then reads the genome Cas9, CRISPR-associated protein 9; PAM, protospacer adjacent motif; sgRNA, single guide RNA Created with BioRender.com; Image credit: Abirami Balasubramanian

In 2012, Dr. Jennifer Doudna and Dr. Emmanuelle Charpentier proposed the use of CRISPR in humans for genome editing (Table 1) [22]. This breakthrough involves combining crRNA and tracrRNA, which are separate entities in bacteria, to form a single molecule with the help of a linker (Figure 3). This synthetically engineered molecule in the lab is called single guide RNA (sgRNA) [22]. This sgRNA is combined with Cas9, sourced from *S. pyogenes*, to form an effector complex, mirroring the natural principle observed in bacteria [20,22]. This innovative approach enables the direct targeting of any 20-nucleotide base pair sequence for editing. By synthesizing an appropriate sgRNA with a sequence complementary to the target sequence and introducing it into the desired cell along with Cas9, the effector complex can read the DNA of the cell until it finds the appropriate sequence along with the PAM sequence, binding to it and precisely cleaving at the desired location (Figure 3) [20-22].

**Table 1:** Summary of findings by Dr. Jennifer Doudna and Dr. Emmanuelle Charpentier from the referenced paper on CRISPR-Cas9 Cas9, CRISPR-associated protein 9; CRISPR, clustered regularly interspaced short palindromic repeats; dCas9, dead Cas9; sgRNA, single guide RNA Source: Charpentier and Marraffini (2014) [22]

Author/year	Name	Topic/focus	Paradigm/method	Context/setting	Findings	Future research
Charpentier and Marraffini (2014)	Harnessing CRISPR-Cas9 immunity for genetic engineering	To understand the mechanism of CRISPR-Cas9 and explore its potential applications as a genome editing tool	Experiment method	The research setting involves laboratory-based studies where CRISPR-Cas9 has been successfully used to achieve desired genetic modifications.	CRISPR is a prokaryotic immune system that functions as a genetic interference mechanism to control the spread of viruses and plasmids. The same CRISPR-Cas system can be repurposed for sequence-specific DNA cleavage. Among the multiple CRISPR-Cas systems, type II systems are considered optimal. These also require a minimal set of components: the Cas9 protein and two RNAs-tracrRNA and crRNA, of which Cas9 is said to perform very efficient DNA cleavage. By engineering a dual tracrRNA:crRNA into a sgRNA, it can form base-pairing with the target DNA sequence and be programmed with Cas9. The double-strand DNA breaks induced by this system are repaired through homologous recombination or non-homologous end joining. Mutations in Cas9’s active site convert it into dCas9, which can bind to promoters to block transcription and repress gene expression. dCas9 can also be fused to activator domains to activate gene expression, although this activation is less efficient than repression. In eukaryotes, dCas9 effectively represses gene expression when linked to repression domains (e.g., KRAB or SID) and less efficiently activates it when linked to activator domains (e.g., VP16/VP64 or p65).	Future developments are expected to include more innovations for gene manipulation, such as fine-tuning dCas9-effector fusions with epigenetic modifiers and recombinases, providing endless opportunities. Research will focus on understanding the various properties of CRISPR-Cas9 systems to enhance the technology further. Additionally, uncovering the nucleic acid-targeting mechanisms of other CRISPR-Cas systems may lead to new biological tools and applications.

**Figure 4:**
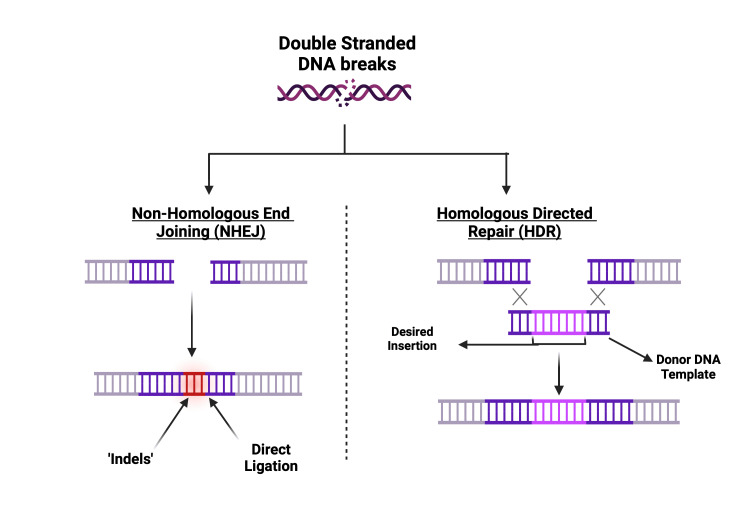
DNA repair via two pathways: NHEJ and HDR HDR, homology-directed repair; NHEJ, non-homologous end joining Created with BioRender.com; Image credit: Abirami Balasubramanian

Cas9 functions as a genetic scissor, comprising two domains, namely recognition (REC) and nuclease (NUC) [17,20,23]. The NUC domain’s subunits will snip each DNA strand, creating double-strand breaks (DSB). Following incision, the natural DNA repair mechanism is activated through two pathways: non-homologous end joining (NHEJ) and homology-directed repair (HDR) (Figure 4) [22]. NHEJ, more prevalent in eukaryotes, repairs the DSB by direct ligation without requiring a homologous template. During this repair, NHEJ is also capable of introducing insertions or deletions of specific sequences, resulting in the formation of “indels” [19,22,23]. Indels are DNA strands that can disrupt protein-coding sequences, resulting in the expression of truncated proteins or no transcription at all. Hence, the outcome of NHEJ is DNA strands with non-uniform strands. On the other hand, the HDR pathway, which is more organized and commonly found in bacteria and archaea, utilizes a homologous template with similar base pairs to the adjacent sequences around the site of cleavage. The REC domain on Cas9 aids in incorporating new DNA fragments into the cleavage site without introducing additions or deletions, leading to a more uniform outcome and a lower error rate [22,24]. HDR predominantly occurs in the S/G2 phase of the mammalian cell cycle and is generally the preferred pathway for precise DNA repair [24,25].

Application of CRISPR-Cas9 in cancer therapeutics

**Figure 5:**
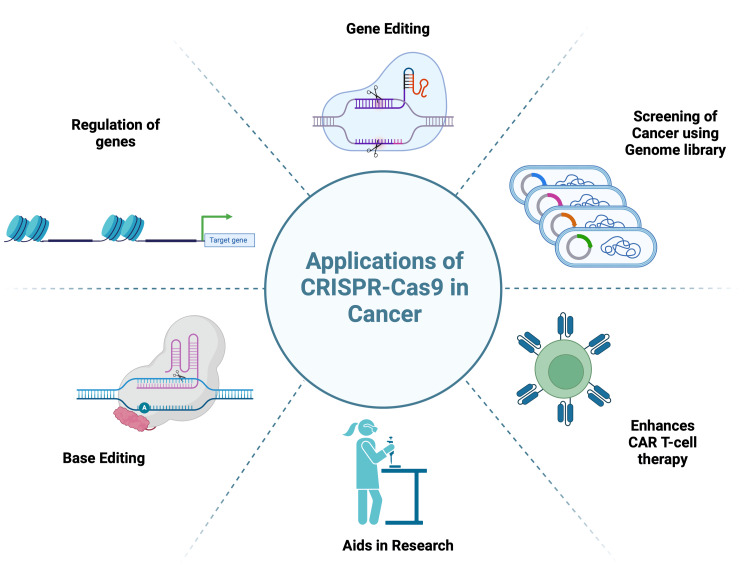
Applications of CRISPR-Cas9 in cancer Cas9, CRISPR-associated protein 9; CRISPR, clustered regularly interspaced short palindromic repeats Created with BioRender.com; Image credit: Abirami Balasubramanian

Cancer treatment can be approached diversely, ranging from surgical debulking and shrinking of tumors with radiotherapy to molecular targeting of tumor-associated genes [26]. At the forefront of molecular therapeutics, CRISPR-Cas9 has attained the pinnacle of precision in modulating gene expression and disrupting cancerous growth at the molecular level [27]. By harnessing the repair mechanisms of CRISPR-induced DNA breaks, researchers can manipulate key genetic pathways involved in tumorigenesis (Figure 5).

*CRISPR Knockout and Identification of Tumor Suppressor Genes* 

**Figure 6:**
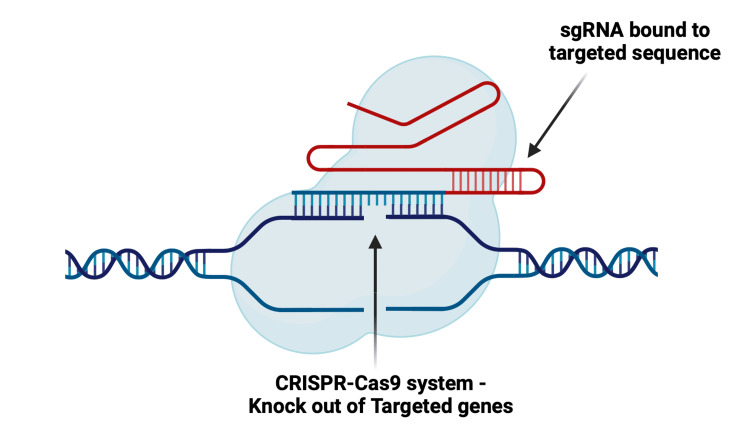
Knockout of targeted genes using CRISPR-Cas9 Cas9, CRISPR-associated protein 9; CRISPR, clustered regularly interspaced short palindromic repeats; sgRNA, single guide RNA Created with BioRender.com; Image credit: Abirami Balasubramanian

**Figure 7:**
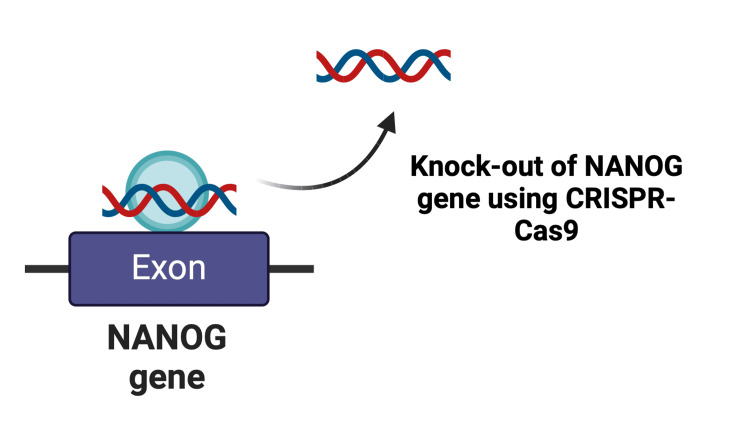
Knockout of the NANOG gene in tumor cells Cas9, CRISPR-associated protein 9; CRISPR, clustered regularly interspaced short palindromic repeats Created with BioRender.com; Image credit: Abirami Balasubramanian

Oncogenes are distinct from normal genes and are vital for either the initiation of oncogenesis or for sustaining the persistence of cancer [28]. The advent of CRISPR-Cas9 directly targets these genes to achieve the elimination of cancer. Upon identifying the specific genes responsible, CRISPR-Cas9 can be applied in vivo to knock out the genes involved (Figures 6, 7) [29]. For example, NANOG is a vital transcription factor associated with the proliferation of cancer cells, with various subtypes [30]. Among them, NANOG1 and NANOG8 are well known to increase tumorigenicity, proliferative power, and drug resistance in prostate cancer [30]. Lu et al. observed that NANOG1 overexpression amplifies invasiveness in breast cancer [31], and it was also discovered by Siu et al. that NANOG1 increases migrative capacity in ovarian cancer [32]. CRISPR-Cas9-mediated knockdown conducted by Yan et al. of MiR-3064 remarkably attenuated the progression and virility of pancreatic cancer [33]. Knockdown of E3 ubiquitin ligase UBR5 resulted in diminished tumor growth and metastasis in triple-negative breast cancer (TNBC) [34]. Innovation in the CRISPR-Cas9 system extends to the detection and validation of tumor suppressor genes, whose silencing can lead to oncogenesis. Studies by Zuckermann et al. have revealed that the deletion of single gene Ptch1 or multiple genes including p53, PTEN, and Nf1 can trigger the development of medulloblastoma and glioblastoma, respectively, thereby validating these candidates as tumor suppressor genes [35]. Furthermore, Wahiduzzaman et al. demonstrated that knocking out the NF2 gene in malignant pleural mesothelioma led to rising levels of fibroblast growth factor receptor 2 (FGFR2), which in turn up-regulated the cloning and migration activity of tumor cells, suggesting FGFR2 as a potential therapeutic target [36]. Moreover, CRISPR can also repair the silenced tumor suppressor genes, offering therapeutic benefits. Moses et al. fused CRISPR with dead Cas9 and transactivator VP64-p65-Rta to increase PTEN expression in melanoma and TNBC cell lines, resulting in a significant reduction of downstream signaling of oncogenic genes such as mTOR and MAPK [37].

CRISPR-Induced Translocations 

**Figure 8:**
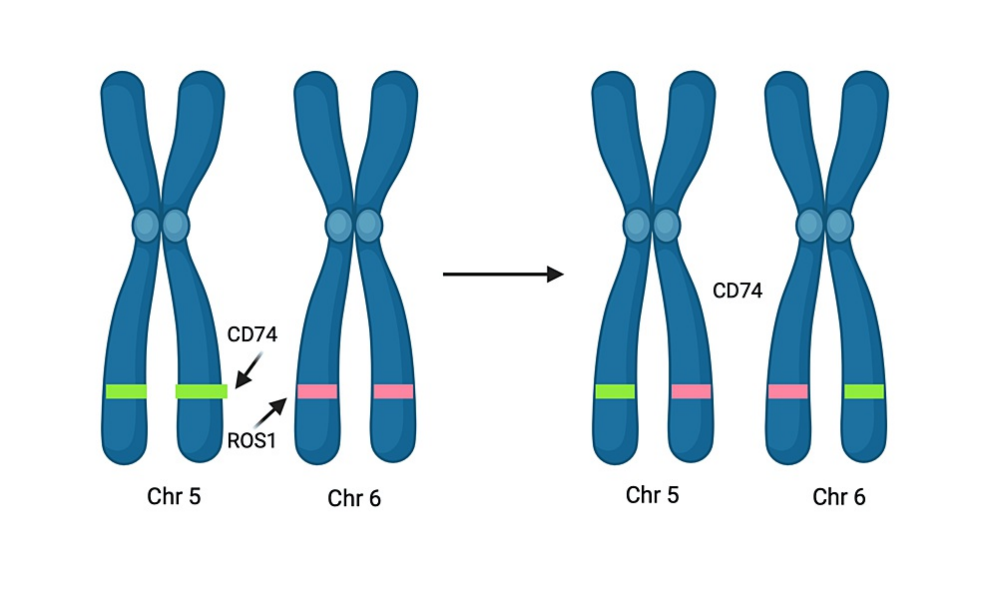
Translocation of CD74 of Chr 5 with ROS1 on Chr 6 using CRISPR Chr, chromosome; CRISPR, clustered regularly interspaced short palindromic repeats Created with BioRender.com; Image credit: Abirami Balasubramanian

**Figure 9:**
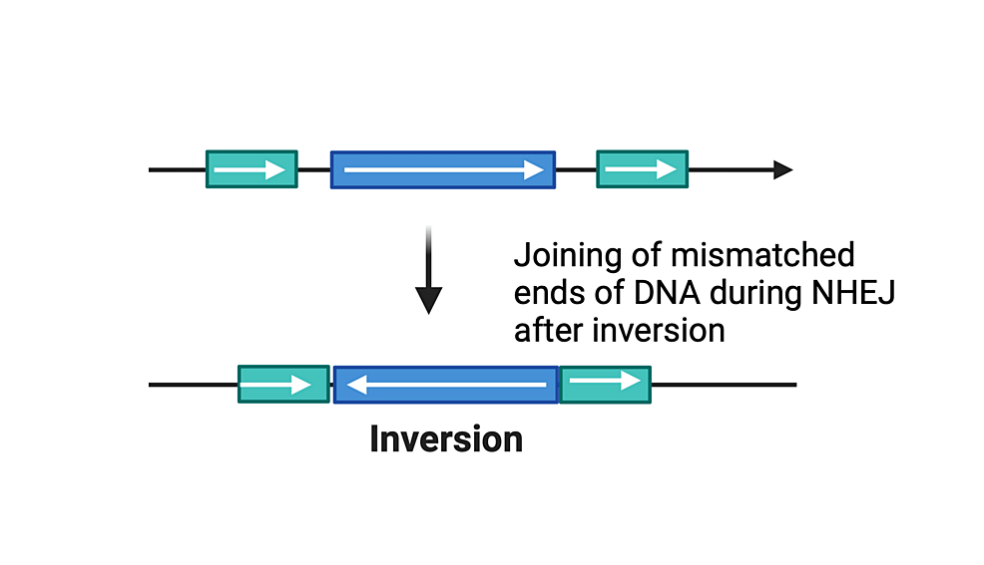
Mismatched ends created by translocations and inversions NHEJ, non-homologous end joining Created with BioRender.com; Image credit: Abirami Balasubramanian

When the Cas9 nuclease induces breaks at two genetic loci simultaneously, it facilitates the possibility of rearrangements such as translocations and inversions [38]. These alterations gradually accumulate over time, promoting malignant changes known as chromothripsis [38]. Better illustrated with an example of lung adenocarcinoma, where translocation of CD74 on chromosome 5 with ROS1 on chromosome 6 is observed (Figure 8) [39]. Similarly, in lung cancer, Cas9 can be utilized to engineer paracentric or pericentric inversions, such as EML4-ALK paracentric rearrangement on chromosome 2 [40,41] and KIF5B-RET pericentric rearrangement on chromosome 10 [40,42], both creating mismatched ends of DNA (Figure 9) [39]. These translocations and inversions are errors that occur during DNA repair [38], predominantly through direct ligation. Thus, the error-prone NHEJ is directly implicated in the fusion of mismatched ends of the DNA [43]. The transcription of these rearrangements may also inactivate many tumor-suppressing genes [44]. CRISPR-Cas9-induced translocations and inversions can aid in deciphering specific genetic alterations involved in oncogenesis and exploring possible therapeutic applications of the same [29,44].

CRISPR as a Screening Tool for Cancer

As already known, the occurrence of cancer is intricately linked to interactions among multiple genes. CRISPR technology can be used to accelerate the identification of these genes and confirm their associated phenotypes, including those responsible for early development, metastasis, metabolic abnormalities of tumor cells, and those accountable for drug resistance [45,46]. By pinpointing these genes, researchers can identify potential therapeutic targets and gain insights into the underlying mechanisms of cancer development, virulence, and resistance to therapy [45,47]. Importantly, not all genotypes that transcribe to cancer phenotypes are solely related to cell proliferation, and some may influence cancer in other ways, which can be deciphered with the use of CRISPR, thereby suggesting alternative treatment modalities. Zhao et al., for instance, utilized a genome-wide CRISPR library to provide evidence that ELAVL2-CDKN1A is involved in chemoresistance in esophageal carcinoma [48]. CRISPR-Cas9 has effectively identified the cancer driver mutation PTEN, which is a negative regulator of the PI3K/AKT signaling pathway in breast, prostate, and endometrial carcinoma [49,50]. When the genes of interest are already identified, a library of corresponding sgRNAs can be created and introduced into suspected cells using CRISPR-Cas to target the specific sites within the genome associated with cancer, thereby confirming its presence [47]. Beyond Cas9, engineered Cas12a and Cas13 enzymes have been instrumental in the rapid detection of tumor DNA and cancer-associated viruses with high precision [24,51]. For instance, Jiang et al. demonstrated the detection of repetitions of Epstein-Barr virus DNA using a CRISPR-Cas12a-mediated amplification-free digital DNA assay, aiding in the screening and diagnosis of nasopharyngeal carcinoma [52]. Similarly, Chen et al. experimented with CRISPR-Cas12a to detect circulating colorectal-associated RNAs (piRNA and miRNA) [53]. Recent advancements and the expansion of genome libraries have facilitated improved high-throughput screening of cancer-related genes and pathways with CRISPR [54]. Shi et al. harnessed CRISPR-Cas9 to precisely mutate certain protein domains within cancer cells, observing diminished growth or clonogenicity as a result [47]. Their study adapted this screening of protein domains to unearth potential cancer drug targets, thereby refining the form of treatment to be provided. The synthesis of multiple genetic barcodes detectable as proteins called “Pro-Codes” overcomes the previous low-dimensional DNA-reliant mechanism, and pairing these with the CRISPR gRNA library has allowed high-resolution screening for breast cancer and recognizing cells resistant to immunotherapy [55,56]. Overall, this functional genetic screening with CRISPR is not just useful for screening cancer but also for other diseases; hence, it is a safe and promising tool.

CRISPR to Mitigate Therapy Resistance in Cancer Cells

CRISPR-Cas9-induced mutagenesis offers a compelling strategy to address treatment resistance in cancer cells [57]. An illustrative example involves germline mutations of the BRCA1 gene (mBRCA1) associated with breast cancer [58,59]. These mutations, especially in exon 11 of the gene, hinder HDR during DSB repair, rendering these cells more sensitive to platinum agents that induce DSBs [58,59]. By utilizing CRISPR-Cas9 to target and delete the PARP1 gene that confers drug resistance, a synergistic effect has been observed in treating tumors harboring mBRCA1 mutations with other anti-cancer drugs [60]. Knockout to induce a missense mutation from CTG to CGG in the EGFR oncogene resulted in increased sensitivity to radiotherapy and prominent DNA damage [61].

CRISPR Insights Into Aging and Tumor Cachexia

The correlation between aging-like genetic abnormalities and susceptibility to colon cancer can be explained with the help of CRISPR. Spontaneous promotor hypermethylation occurs in certain cells, regulating aging processes [62]. Utilizing CRISPR to inactivate these genes associated with the control of aging activates the Wnt pathway, promoting the proliferation of stem cells. This, in turn, makes the cells more susceptible to the Braf V6000E mutation and the development of colon cancer [63]. Thus, it highlights old age as a poor prognostic factor. Likewise, tumor cachexia contributes to a dismal prognosis. Upon identifying the gene initiating cancer cachexia, Kandarian et al. experimented with CRISPR-Cas9 knockout to target leukemia inhibitory factor, ameliorating muscle wasting and improving cancer outcome [64].

CRISPR Disruption of Metabolism

As a characteristic property of tumor cells, they exhibit the Warburg effect, defined by heightened consumption of glucose compared to normal cells. A double knockout of lactate dehydrogenase A and lactate dehydrogenase B can be achieved with CRISPR, which then effectively inhibits the glycolytic pathway essential for cancer survival [65]. This approach not only aids in understanding the metabolic pathways involved but also targets cancer by curtailing oxidative metabolism [65]. Similarly, CRISPR has been used to strategically target OXPHOS, which is responsible for the persistence of cancer cells in acidic pH environments. Combining this with anti-vascular endothelial growth factor therapy to induce hypoxia creates an acidic microenvironment, thereby synergistically killing cancer cells [66].

CRISPR in CAR-T Cell Therapy

As previously mentioned, immunotherapy is a well-known treatment modality for cancer, with CAR T-cell therapy being an example [67]. Typically, T-cells mount a normal immune response against tumor cells. The latter, however, can mask themselves from recognition by reducing the number of MHC1 on their surface, which is responsible for attracting killer T-cells. T-cells deployed tend to exhaust soon due to the continued presence of inhibitory receptors, the most remarkable of which are PD-1 and CTLA-4. On binding to complementary ligands, namely PD-L1/L2, on tumor cells, the respective T cells are inactivated and hence exhausted. CTLA-4 is an inhibitory co-receptor, and its expression on T-regulatory cells enhances their immunosuppressive function [68]. In CAR T-cell therapy, to improve recognition, the patient’s T cells are harvested and reprogrammed to express chimeric antigen receptors, which enables them to effectively recognize and latch onto the tumor cells independent of MHC1 [67]. The signaling domain within CAR-T cells triggers the destruction of tumor cells through cytokine release, exponentially decreasing the tumor burden and being particularly effective against hematological malignancies like leukemia [69] and multiple myeloma [70]. CRISPR can be used to augment the response created by these CAR-T cells by prolonging activity and preventing exhaustion of CAR-T cells [67]. Knocking out the PD-1 receptor gene effectively averts depletion and prolongs T-cell action against tumor cells [71]. Similarly, knocking out the gene responsible for the production of PTP1B in T-cells has also been shown to enhance the anti-tumor activity of CAR-T cells against solid tumors. PTPB1 serves as a negative regulator that dampens T-cell responses and reinforces PD-1 blockade [67]. Likewise, Tang et al. knocked out the TGFBR2 gene in CAR-T cells, thereby removing negative regulation of transforming growth factor beta-1 and improving immune surveillance of T cells [72]. Disruption of CTLA-4 through knockout may further potentiate the CAR-T cell response by inhibiting its suppressive effects on T cells [73]. Excessive cytokine release, particularly of GM-CSF, by monocytes and macrophages can induce cytokine release syndrome and neurotoxicity, which may further result in premature inactivation of CAR-T cells [74]. CRISPR-mediated knock-out of GM-CSF amplifies the anti-tumor activity of CAR-T cells. Patients who have undergone multiple cycles of chemotherapy, as well as elderly individuals and neonates, are not ideal candidates for CRISPR-CAR-T cell therapy due to their low reserve of T cells [67]. As an efficient gene editing tool, CRISPR has significantly ameliorated the use of one’s T cells to combat cancer.

CRISPR Base Editing

**Figure 10:**
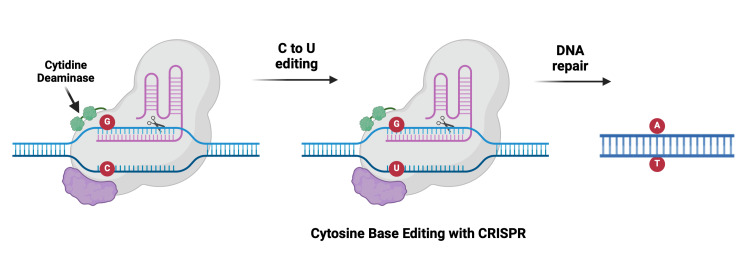
Cytosine base editing with CRISPR CRISPR, clustered regularly interspaced short palindromic repeats Created with BioRender.com; Image credit: Abirami Balasubramanian

CRISPR technology components can be used in the latest genome editing techniques called cytosine base editors (CBE) and adenine base editors (ABE). CBE facilitates the direct conversion of a C-G base pair to an A-T base pair (Figure 10), while ABE converts A-T to C-G, obtaining precise point mutations without inducing any DSBs [75]. By doing so, we can introduce premature “stop” codons, hence their use in cancer therapeutics. This method, as described by Komor et al., is an irreversible conversion and minimizes off-target effects, thus ensuring safety and reliability. Its practical application includes correcting the p53 mutation in breast cancer [76].

Advantages of CRISPR-Cas9

Nucleases used in genome editing include zinc-finger nucleases (ZFNs), transcription activator-like effector nucleases (TALENs), and CRISPR-Cas9, with the former two relying on DNA-protein interaction. CRISPR offers several advantages over these methods, the primary advantage being its high target specificity, with the only requirement being the presence of a PAM sequence for efficient targeting [77]. Any sort of mutation induced by CRISPR is sufficient to disrupt the target genome, proving its versatility [78]. Customization of CRISPR to accommodate specific needs is possible. For example, using larger CRISPR arrays can generate multiple crRNAs, enabling simultaneous targeting of multiple sites and affecting the orchestrated interactions among these genes [78]. Achieving the same customization in ZFNZs and TALENs has been challenging to achieve. CRISPR is also more efficient, as modifications can be directly injected into target cells with sgRNA [78,79]. Unlike ZFNs and TALENs, which are time-consuming, labor-intensive, and require protein engineering, CRISPR offers a more streamlined process. Cas9 could also be tailored by creating variants like Cas9 nickase (Cas9n) that direct DSB preferentially to high-fidelity HDR, enhancing specificity. The design of CRISPR is easier and more cost-efficient to construct compared to that of meganucleases and ZFNs [79]. Off-target effects are a concern for all genome editing techniques; likewise, the on/off-target ratio is not well defined for ZFNs and TALENs and can result in toxicity. Although off-targets are inevitable even while using CRISPR, it facilitates easy prediction of the type of off-targets and offers more control to minimize them effectively compared to other methods [29]. Hence, CRISPR-Cas9 genome editing is considered superior to the other forms, namely, ZFN and TALEN.

Limitations and challenges

Complexity of the DNA Repair Mechanism

**Figure 11:**
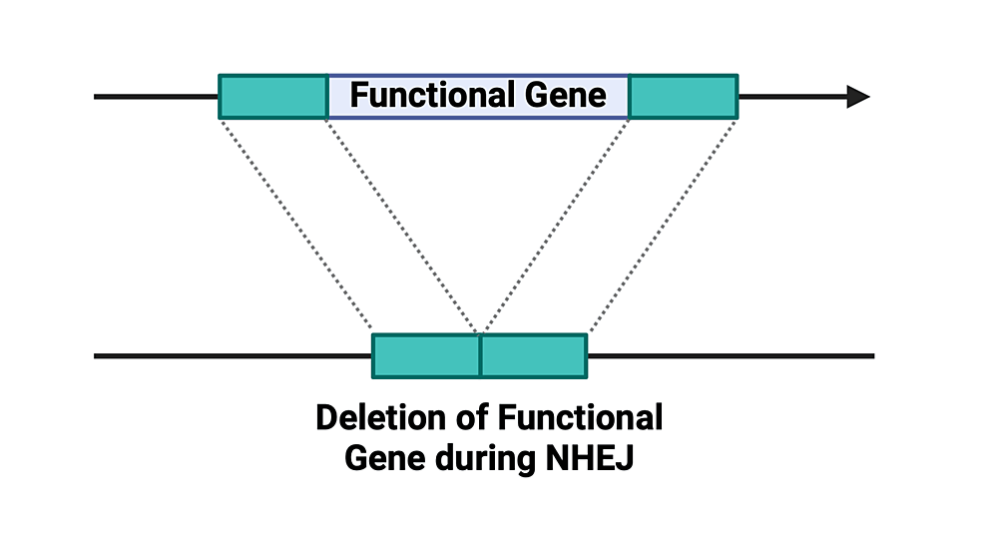
NHEJ-induced deletion of functional gene NHEJ, non-homologous end joining Created with BioRender.com; Image credit: Abirami Balasubramanian

**Figure 12:**
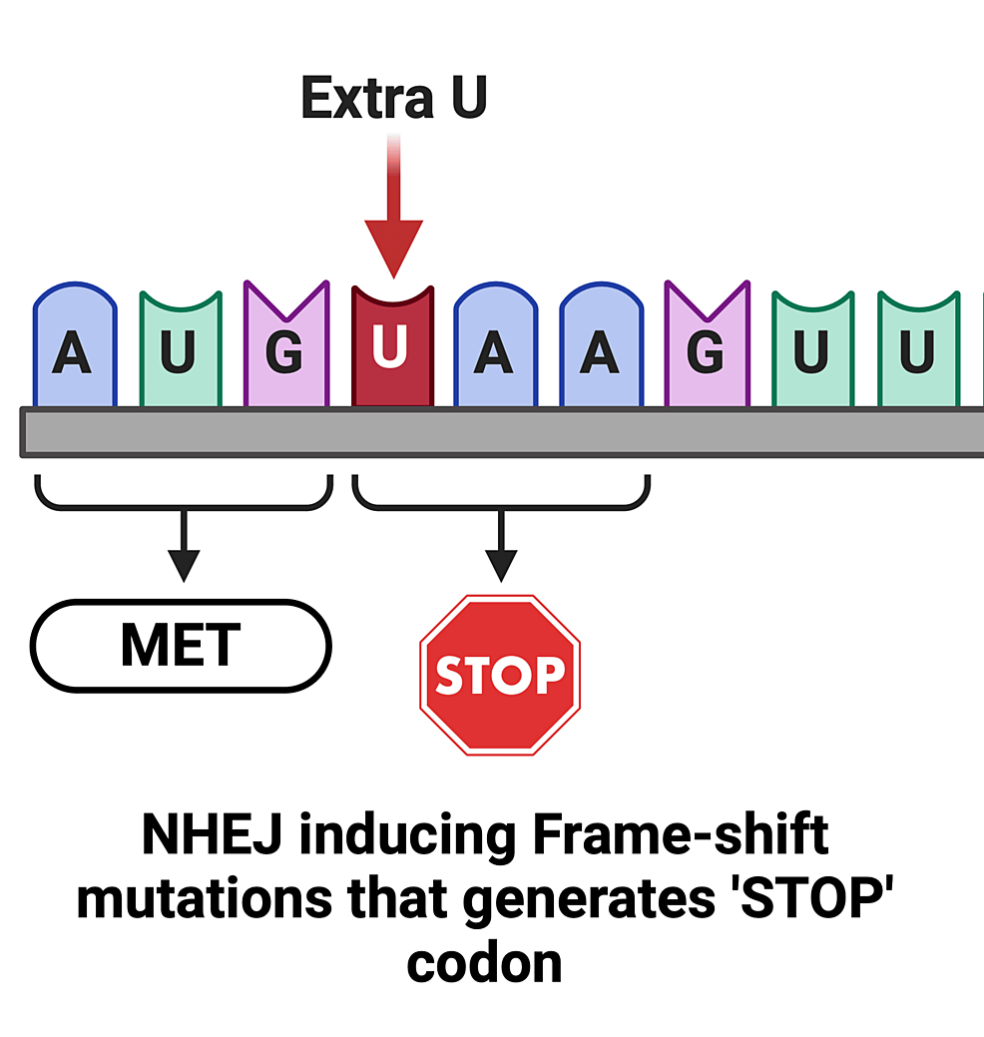
Frameshift mutation in NHEJ generating the ‘STOP’ codon NHEJ, non-homologous end joining Created with BioRender.com; Image credit: Abirami Balasubramanian

As previously mentioned, DNA repair following a double-stranded break occurs through two distinct pathways: NHEJ and HDR. NHEJ is a spontaneous and naturally occurring mechanism for gene correction, often considered more efficient [23]. However, due to its predisposition to form insertions or deletions (indels), NHEJ poses a risk of frameshift mutations that may result in the generation of stop codons or the coding of truncated proteins with impaired functionality (Figures 11, 12). These unintended mutations could potentially have detrimental effects, complicating therapeutic efforts in the treatment of cancer [19,22,23]. On the other hand, HDR is characterized by greater specificity and is generally more preferred. To favor HDR over NHEJ, the inhibitor Scr7 can be used, which acts against the key enzyme of NHEJ, DNA Ligase IV [80]. By inhibiting NHEJ, the likelihood of HDR is enhanced. While HDR provides a more controlled and accurate repair process, it is less efficient compared to the error-prone NHEJ and may not be as suitable for large-scale production [25]. The precision of HDR, too, is contingent on the administration of an appropriate homologous donor template for gene editing, necessitating efforts to engineer such a template. Research conducted by Ran et al. demonstrated that replacing Cas9 with Cas9n enhances efficiency and reduces off-target effects and indel formation for both NHEJ and HDR [81].

Delivery of CRISPR-Cas9 to Cells

An inherent challenge lies in the potent delivery of the CRISPR-Cas9 system to tumor cells, which is key for accurately targeting all cancerous cells. Currently, there are various methods like the use of viral vectors, injection, vesicles, and nanoparticles, each of which has its drawbacks related to safety, efficacy, and financial feasibility [82,83]. Viral vectors, for instance, may pose risks such as immunogenicity and cytogenicity. They may even carry the potential of being integrated into the host genome, particularly in lentiviruses. Although adeno-associated viruses are commonly used due to their low disease-causing potential in humans, they have limited packaging capacity, making them incapable of carrying larger Cas variants like SpCas. Adenoviral vectors have shown promise in eliminating off-target effects but may also induce immune reactions [82-84]. Adenoviruses are highly specific and more effective; however, directly injecting these viral vectors can induce liver damage. Nevertheless, reducing the immunogenicity of viral vectors for efficient use includes high production costs and labor-intensive processes [83,84]. New approaches are being engineered to overcome these barriers. Nonviral vectors, such as cationic lipids and peptide nanoparticles, offer higher loading capacity, lower immunogenicity, and counteract the risk of endogenous viral integration [67,84]. Extracellular vesicles, particularly exosomes, which are lipid-coated particles, are considered most genuine due to their close resemblance to tumor cells and ease of customization [84]. However, it is vital to note that nonviral vectors are incomparable to viral vectors in terms of efficiency and potency.

“Off-Target” Effects

The prevalence of off-target effects is a substantial concern for CRISPR-Cas9 as a therapeutic application, as its specificity is not perfect. While the sgRNA is designed to introduce indels at specific target sites, there is a risk of it being misdirected, leading to similar genetic alterations in regions other than the target site [29]. Such unintended consequences, instead of addressing the intention to treat cancer, may worsen the plight by inducing additional mutations and deletions or by producing truncated proteins, thereby predisposing them to other diseases and deteriorating their health condition. Strategies to alleviate off-target effects include modifying sgRNA to target shorter complementary DNA sequences of less than 20 nucleotides and reducing the duration of sgRNA expression and binding [29,85]. Both of these narrow targeting measures help improve the on-off target ratio. Moreover, genetic engineering has enabled the production of different variants of Cas proteins, allowing control over the period of active Cas9 generation and hence reducing excessive activity of CRISPR-Cas9 in the system, thereby minimizing off-target effects [29,45,86]. Additionally, it has been reported that excess DNA binding energy increases off-target gene editing. By introducing mutations into Cas9, this energy can be neutralized, reducing nonspecific alterations [29,85]. All these actions collectively improve the specificity of the CRISPR-Cas9 system.

“Escapers” of CRISPR-Cas9 Gene Editing

A recently discovered limitation is the formation of “escapers,” a population of cells that escape CRISPR gene editing. The predominant mechanism for this has been found to involve the deletion of the targeting spacer, enabling cells to evade targeted cleavage by Cas while preserving the normal functions of the cell [87]. In addition, mutations in the PAM sequence, essential for recognizing the complementary DNA strand, can also hinder effective targeting [88]. Consequently, attempts to genetically edit these cells to eliminate the tumorigenic mutations would end in failed efforts. The evolution of phages to develop resistance against bacteria led to the development of a naturally occurring group of peptides known as anti-CRISPR proteins, which inhibit CRISPR-Cas, allowing them to evade being targeted as well [89]. This potential can be used to our benefit, as these proteins can serve as an “off-switch” for CRISPR, enabling control and restriction of its activity to specific tissues, thereby reducing off-target effects [90].

Ethical Considerations

Although CRISPR-Cas9 represents a remarkable leap in human innovation and scientific advancement, it also raises profound ethical concerns regarding the manipulation of natural processes and their fundamental laws [91]. There has always been a strong public backlash, with many arguing that despite its holistic approach, CRISPR violates the sanctity of humanity, citing moral arguments against researchers “playing God” [92]. Recent episodes of experimenting with human embryos in China have intensified the ethical debate, prompting the country to revise and strengthen its ethical guidelines and regulations [93]. This is due to the potential for germline editing to be passed onto successive generations, given the permanent nature of CRISPR-Cas9 editing. Although the same in cancer therapeutics is somatic editing, similar concerns arise due to the limited public understanding of the theory and long-term implications of CRISPR for cancer treatment and prevention [91]. The limitless possibility, feasibility, and potential misuse of CRISPR may inadvertently breach the three principles of bioethics, resulting in “nonmaleficence” [94]. The growth of genome editing technologies is thus constrained by the adverse public response and unclear regulatory policies. In light of these ethical uncertainties, achieving a balance between scientific progress and reverence for the sanctity of the natural order becomes pivotal.

## Conclusions

CRISPR-Cas9 marks the pinnacle of success in genetic engineering. Initially discovered as a prokaryotic defense mechanism, its profound impact on human health, particularly in the field of oncology, has been transformative. The ease of customization, feasibility, and fidelity of CRISPR establish its superiority. Concerns such as off-target effects remain before it can be widely embraced as a standard cancer treatment. Despite these challenges, the primary purpose of CRISPR-Cas9 is to alleviate human suffering while maintaining safety, dignity, and adherence to bioethical principles. The potential benefits outweigh the challenges involved, and continuous efforts are required to overcome the limitations and realize its full potential. With ongoing human innovation, CRISPR holds promise as a clinical treatment and a powerful tool for preventing and treating genetic diseases, including cancer.
